# From arteries to boreholes: steady-state response of a poroelastic cylinder to fluid injection

**DOI:** 10.1098/rspa.2016.0753

**Published:** 2017-05-31

**Authors:** L. C. Auton, C. W. MacMinn

**Affiliations:** Department of Engineering Science, University of Oxford, Oxford OX1 3PJ, UK

**Keywords:** porous media, poroelasticity, nonlinear elasticity, spectral collocation

## Abstract

The radially outward flow of fluid into a porous medium occurs in many practical problems, from transport across vascular walls to the pressurization of boreholes. As the driving pressure becomes non-negligible relative to the stiffness of the solid structure, the poromechanical coupling between the fluid and the solid has an increasingly strong impact on the flow. For very large pressures or very soft materials, as is the case for hydraulic fracturing and arterial flows, this coupling can lead to large deformations and, hence, to strong deviations from a classical, linear-poroelastic response. Here, we study this problem by analysing the steady-state response of a poroelastic cylinder to fluid injection. We consider the qualitative and quantitative impacts of kinematic and constitutive nonlinearity, highlighting the strong impact of deformation-dependent permeability. We show that the wall thickness (thick versus thin) and the outer boundary condition (free versus constrained) play a central role in controlling the mechanics.

## 1. Introduction

The radially outward flow of fluid into a porous medium is central to many practical problems in, for example, geomechanics, biophysics and filtration. In geomechanics, pile driving involves the mechanical expansion of a cylindrical cavity in a fluid-saturated soil, generating large pore pressures in the surrounding medium that gradually relax through consolidation [1]. Similarly, fluid injection into boreholes involves the pressurization of a cylindrical cavity in a soil or rock, driving flow radially outward into the surrounding medium [2–5]. Biophysical applications include injection into subcutaneous tissue [6] and blood flow through arteries and vascular networks, which have permeable walls [7–13]. Radially outward flow is also relevant to the design of cylindrical filters [14]. In many of these cases, the driving pressure is sufficiently large relative to the stiffness of the solid structure that the poromechanical coupling between the fluid and the solid has an important impact on the flow. Classically, this coupling is described by the iconic theory of linear poroelasticity [15,16], which combines Darcy’s Law with linear elasticity in a linearized kinematic framework and is valid for infinitesimal deformations of the solid. However, soft materials such as biological tissues, weak materials such as soils, thin structures such as vasculature, and scenarios involving large injection pressures such as hydraulic fracturing may result in substantial deformations that violate this linear theory. Large deformations are inherently nonlinear from the perspective of kinematics, and typically also result in nonlinear constitutive behaviour such as nonlinear elasticity and deformation-dependent permeability. Recent work in biomechanics and geomechanics, in particular, has focused on capturing the complex material- and application-specific behaviours of tissues and soils [17–23].

Our goal here is to focus on the mechanics of large radial deformations in the context of a simple model problem. We work with relatively generic constitutive laws to avoid obscuring the universal physics of these problems with material-specific behaviour. Historically, uniaxial deformation has been a key model problem for studying the importance of nonlinearity, both mathematically and experimentally [24–27]. The uniaxial problem is important for a variety of practical applications; for example, many composite manufacturing processes involve the uniaxial injection of a resin gel or metal melt into a deformable porous matrix [28]. Mathematically, the uniaxial problem is inherently simple since the flow and deformation fields are strictly one dimensional and the exact relationship between displacement and porosity is linear [27]. Radial deformations are more challenging despite the fact that the velocity and displacement fields remain one dimensional, since the stress and strain fields become biaxial and the exact relationship between the porosity and displacement becomes nonlinear.

Radial poroelastic deformations have been studied using linear poroelasticity in the context of both fluid injection or extraction from boreholes [2–4] and arterial blood flow [7,8]. Nonlinear effects have attracted interest primarily in the latter case, specifically in the context of fluid flow through artery walls. For example, Klanchar & Tarbell [9] introduced deformation-dependent permeability within a linear poroelastic framework. Barry & Aldis [10] and Barry & Mercer [11] accounted partially for the nonlinear kinematics of large deformations while retaining linear elasticity. In a different context, MacMinn *et al.* [29] developed a rigorous and fully nonlinear model, but for a strictly volumetric constitutive law and assuming constant permeability. None of these previous works explicitly defined or explored the general parameter space for axisymmetric deformations, nor did they systematically assess the relative importance of nonlinear kinematics, nonlinear elasticity and deformation-dependent permeability.

Here, we consider the axisymmetric deformation of a poroelastic cylinder driven by radially outward fluid flow using a rigorous, fully nonlinear model. We focus, in particular, on the qualitative and quantitative implications of the simplifications of linear poroelasticity, the separate roles of nonlinear kinematics, nonlinear elasticity and deformation-dependent permeability, and the nontrivial coupling of these with the geometry and boundary conditions. We show that the wall thickness and the outer boundary condition play crucial roles in controlling the mechanics of the problem.

## 2. Model problem

**Figure 1.**
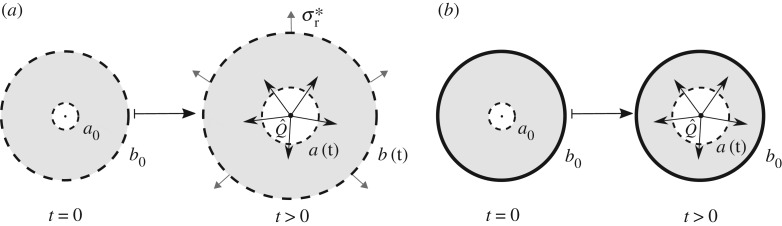
We consider radially outward fluid flow through a soft porous cylinder of initial inner radius *a*_0_ and initial outer radius *b*_0_. The inner radius is free to expand, while the outer boundary is either (*a*) subject to a constant radial effective stress $\sigma_{r}^{⋆}$ or (*b*) fixed in place. Note that we assume plane strain and adopt the convention of tension being positive.

We consider the radially outward injection of fluid from the centre of a porous cylinder of inner radius *a* and outer radius *b*. We assume axisymmetry and model the two-dimensional annular cross-section, assuming that the material is constrained in the axial direction and is therefore in plane strain. We assume that the inner boundary is mechanically free so that the inner radius *a*=*a*(*t*) expands in response to injection. We assume that the outer boundary is either subject to a constant effective stress $\sigma_{r}^{⋆}$, in which case the outer radius *b*=*b*(*t*) also expands in response to injection (figure 1*a*), or that the outer boundary is constrained such that the outer radius *b*=*b*_0_ is fixed (figure 1*b*). The latter situation is useful for comparison to numerical simulations and experiments [29].

### (a) Summary of theory

Large-deformation poroelasticity is a continuum approach to modelling the interactions of two superposed phases, a porous solid skeleton and an interstitial fluid [27]. We next summarize this theory in the context of axisymmetric flow and deformation.

#### (i) Kinematics

The fluid velocity **v**_*f*_, solid displacement **u**_s_ and solid velocity **v**_s_ each have only one component,

2.1 $${\text{u}}_{s}=u_{s}(r,t){\hat{\text{e}}}_{r},\;{\text{v}}_{s}=v_{s}(r,t){\hat{\text{e}}}_{r}\;\text{and}\;{\text{v}}_{f}=v_{f}(r,t){\hat{\text{e}}}_{r},$$

where the subscripts s and f denote quantities related to the solid and to the fluid, respectively, *r* is the radial coordinate (*a*≤*r*≤*b*), *t* is time and ${\hat{\text{e}}}_{r}$ is the radial unit vector. We work in an Eulerian (spatial) reference frame, such that the displacement is given by

2.2 $$u_{s}(r,t)=r-R(r,t),$$

where *R*(*r*,*t*) denotes the reference position of the material that is located at position *r* at time *t*. Without loss of generality, we take *u*_s_(*r*,0)=0 such that *R*(*r*,0)=*r*—that is, we adopt the initial configuration as the reference configuration. The deformation is fully characterized by the deformation gradient tensor **F**=(**I**−∇**u**_s_)^−1^, where **I** denotes the identity tensor and (⋅)^−1^ the inverse. For an axisymmetric deformation, this can be written as

2.3 $$\text{F}=\left(\begin{array}{ccc} \lambda_{r} & 0 & 0\\ 0 & \lambda_{\theta } & 0\\ 0 & 0 & \lambda_{z} \end{array}\right),$$

where λ_*r*_, λ_*θ*_ and λ_*z*_ are the three principal stretch ratios.^1^ For plane strain, these are given by

2.4 $$\lambda_{r}={\left(1-\frac{\partial u_{s}}{\partial r}\right)}^{-1},\;\lambda_{\theta }={\left(1-\frac{u_{s}}{r}\right)}^{-1}\;\text{and}\;\lambda_{z}\equiv 1.$$

Note that although the displacement field is one dimensional, the state of strain is indeed two dimensional (i.e. both λ_*r*_ and λ_*θ*_ are distinct and non-trivial).

The Jacobian determinant J≡det (F) measures the local volume change,

2.5 $$J=\lambda_{r}\lambda_{\theta }\lambda_{z}=\lambda_{r}\lambda_{\theta }.$$

We assume that the solid and fluid phases are individually incompressible, such that deformation occurs only through rearrangement of the solid skeleton with corresponding changes in the local porosity or fluid fraction, *ϕ*_f_. This then requires that

2.6 $$J(r,t)=\frac{1-\phi_{f,0}}{1-\phi_{f}},$$

where *ϕ*_f,0_ is the reference (initial) porosity, which we take to be uniform. Combining equations (2.4)–(2.6), we obtain an explicit nonlinear expression for porosity in terms of displacement,

2.7 $$\frac{\phi_{f}-\phi_{f,0}}{1-\phi_{f,0}}=\frac{1}{r}\frac{\partial }{\partial r}\left(ru_{s}-\frac{1}{2}u_{s}^{2}\right).$$

Conservation of mass for the fluid–solid mixture is given by

2.8 $$\frac{\partial \phi_{f}}{\partial t}+\frac{1}{r}\frac{\partial }{\partial r}(r\phi_{f}v_{f})=0\;\mathrm{and}\;\frac{\partial \phi_{f}}{\partial t}-\frac{1}{r}\frac{\partial }{\partial r}[r(1-\phi_{f})v_{s}]=0,$$

where 1−*ϕ*_f_ is the local solid fraction. Conservation of solid volume requires that

2.9 $$\int_{a}^{b}2\pi r(1-\phi_{f})dr=\pi (b_{0}^{2}-a_{0}^{2})(1-\phi_{f,0}),$$

and it can be shown that equation (2.9) is identically satisfied by equation (2.7), subject to the kinematic boundary conditions *u*_s_(*a*,*t*)=*a*(*t*)−*a*_0_ and *u*_s_(*b*,*t*)=*b*(*t*)−*b*_0_, where *a*_0_≡*a*(0) and *b*_0_≡*b*(0) denote the initial inner and outer radii, respectively.

#### (ii) Darcy’s Law

We assume that fluid flows relative to the solid skeleton according to Darcy’s Law. In the absence of gravity and other body forces, this can be written

2.10 $$\phi_{f}(v_{f}-v_{s})=-\frac{k(\phi_{f})}{\mu }\frac{\partial p}{\partial r},$$

where *μ* is the dynamic viscosity of the fluid, *p* is the fluid (pore) pressure and *k*(*ϕ*_f_) is the permeability, which we take to be an isotropic function of porosity (see §2c).

We model injection as a line source at the origin with flow rate per unit length $\hat{Q}(t)$. Thus, equations (2.8) can be summed and integrated to give

2.11 $$2\pi r[\phi_{f}v_{f}+(1-\phi_{f})v_{s}]=\hat{Q}(t).$$

Combining equation (2.11) with equations (2.8) and (2.10), we eliminate *ϕ*_s_, *v*_s_ and *v*_f_ to obtain

2.12*a* $$\frac{\partial \phi_{f}}{\partial t}+\frac{1}{r}\frac{\partial }{\partial r}\left(\phi_{f}\frac{\hat{Q}(t)}{2\pi }-r(1-\phi_{f})\frac{k(\phi_{f})}{\mu }\frac{\partial p}{\partial r}\right)=0,$$

where along the way we obtain expressions for *v*_f_ and *v*_s_

2.12*b* $$v_{f}=\frac{\hat{Q}(t)}{2\pi r}-\frac{\left(1-\phi_{f}\right)}{\phi_{f}}\frac{k(\phi_{f})}{\mu }\frac{\partial p}{\partial r}\;\mathrm{and}\;v_{s}=\frac{\hat{Q}(t)}{2\pi r}+\frac{k(\phi_{f})}{\mu }\frac{\partial p}{\partial r}.$$

We next link the fluid pressure to the stress in the solid.

#### (iii) Mechanical equilibrium

Mechanical equilibrium requires that

2.13 ∇⋅σ=0,

where ***σ*** is the total stress supported by the fluid–solid mixture, and we neglect inertia as well as the effect of gravity and other body forces. The total stress can be decomposed as

2.14 $$\sigma =\sigma^{\prime }-p\text{I},$$

where Terzaghi’s effective stress ***σ***′ is the portion of the stress supported through deformation of the solid skeleton, and where we adopt the convention of tension being positive. Equation (2.14) provides mechanical coupling between the fluid and the solid. Combining equations (2.13) and (2.14) leads, for an axisymmetric deformation, to

2.15 $$\frac{\partial \sigma_{r}^{\prime }}{\partial r}+\frac{\sigma_{r}^{\prime }-\sigma_{\theta }^{\prime }}{r}=\frac{\partial p}{\partial r},$$

where *σ*′_*r*_ and *σ*′_*θ*_ are the radial and azimuthal (hoop) components of the effective stress, respectively.

#### (iv) Linearization

We have now considered kinematics, Darcy’s Law, Terzaghi’s effective stress and mechanical equilibrium. The model thus far is exact, assuming only that the fluid and solid constituents are individually incompressible.

The common assumption of infinitesimal deformations leads to classical linear poroelasticity [16,27]. This corresponds here to the assumptions that *u*_s_/*r*≪1 and ∂*u*_s_/∂*r*≪1. Note that this will clearly be a bad assumption near the inner radius if *u*_s_ becomes comparable with *a*_0_. Linearizing equations (2.7) and (2.12a) leads to

2.16 $$\frac{\phi_{f}-\phi_{f,0}}{1-\phi_{f,0}}\approx \frac{1}{r}\frac{\partial }{\partial r}(ru_{s})\;\mathrm{and}\;\frac{\partial \phi_{f}}{\partial t}-\frac{1}{r}\frac{\partial }{\partial r}\left(r(1-\phi_{f,0})\frac{k(\phi_{f,0})}{\mu }\frac{\partial p}{\partial r}\right)\approx 0,$$

respectively. Note that equation (2.9) is not identically satisfied by the kinematic expression in equation (2.16), implying that the linearized model is not rigorously mass conservative. We also consider a linearised model with deformation-dependent permeability by simply replacing *k*(*ϕ*_f,0_) with *k*(*ϕ*_f_) in equation (2.16) above. We next consider the constitutive behaviour of the solid.

### (b) Constitutive laws

The relationships between stress and strain, and between strain and displacement are constitutive laws for the solid skeleton. We assume that the solid deforms elastically, meaning that these relationships are quasi-static (i.e. rate independent) and reversible (i.e. history independent). We investigate the impact of this relationship on the results by considering both linear and nonlinear elasticity laws.

#### (i) Hencky elasticity

Hencky elasticity is a simple, nonlinear, hyperelastic model that is based on a logarithmic strain measure and provides good agreement with experiments for moderate deformations [30,31]. In uniaxial compression, Hencky elasticity provides a stiffer response than linear elasticity, with the stress diverging as the thickness of the material approaches zero; in uniaxial tension, Hencky elasticity provides a softer response than linear elasticity, with the stress reaching a maximum and then decaying asymptotically to zero (see electronic supplementary material, section A).

Hencky elasticity has several advantageous properties [32], including that it reduces to linear elasticity in the limit of infinitesimal deformations and that it uses the same elastic parameters as linear elasticity [33]. We work here in terms of Lamé’s first parameter *Λ* and the *p*-wave or oedometric modulus M.

For the displacement field given in equation (2.2), the Hencky strain tensor is

2.17 $$\varepsilon =\left[\begin{array}{ccc} \ln\lambda_{r} & 0 & 0\\ 0 & \ln\lambda_{\theta } & 0\\ 0 & 0 & 0 \end{array}\right],$$

which again has two non-trivial components since axisymmetric displacement leads to both radial and azimuthal strains. The associated Cauchy effective stress for Hencky elasticity is

2.18 $$\sigma^{\prime }=\left[\begin{array}{ccc} M\frac{\ln\lambda_{r}}{J}+\Lambda \frac{\ln\lambda_{\theta }}{J} & 0 & 0\\ 0 & \Lambda \frac{\ln\lambda_{r}}{J}+M\frac{\ln\lambda_{\theta }}{J} & 0\\ 0 & 0 & \Lambda \left(\frac{\ln\lambda_{r}+\ln\lambda_{\theta }}{J}\right) \end{array}\right].$$

On substitution of equation (2.18) into equation (2.15), we arrive at

2.19 $$\frac{\partial p}{\partial r}=\frac{\partial }{\partial r}\left(M\frac{\ln\lambda_{r}}{J}+\Lambda \frac{\ln\lambda_{\theta }}{J}\right)+\frac{M-\Lambda }{r}\left(\frac{\ln\lambda_{r}}{J}-\frac{\ln\lambda_{\theta }}{J}\right).$$

The right-hand side of equation (2.19) is a function of *u*_s_ only. In combination with equations (2.7) and (2.12a), this then provides a nonlinear partial differential equation (PDE) for *u*_s_.

#### (ii) Linear elasticity

Linear elasticity combines a linear relationship between strain and displacement with a linear relationship between stress and strain. The linear (small or infinitesimal) strain tensor is

2.20 $$\varepsilon =\left[\begin{array}{ccc} \frac{\partial u_{s}}{\partial r} & 0 & 0\\ 0 & \frac{u_{s}}{r} & 0\\ 0 & 0 & 0 \end{array}\right]$$

with the associated linear stress tensor

2.21 $$\sigma^{\prime }=\left[\begin{array}{ccc} M\frac{\partial u_{s}}{\partial r}+\Lambda \frac{u_{s}}{r} & 0 & 0\\ 0 & \Lambda \frac{\partial u_{s}}{\partial r}+M\frac{u_{s}}{r} & 0\\ 0 & 0 & \Lambda \left(\frac{\partial u_{s}}{\partial r}+\frac{u_{s}}{r}\right) \end{array}\right].$$

On substitution of equation (2.21) into equation (2.15), we obtain

2.22 $$\frac{\partial p}{\partial r}=\frac{\partial }{\partial r}\left[M\frac{\partial u_{s}}{\partial r}+\Lambda \frac{u_{s}}{r}\right]+\frac{M-\Lambda }{r}\left(\frac{\partial u_{s}}{\partial r}-\frac{u_{s}}{r}\right)=M\frac{\partial }{\partial r}\left[\frac{1}{r}\frac{\partial }{\partial r}(ru_{s})\right].$$

Linear elasticity is in some sense an idealized constitutive behaviour that most materials will approximately follow for infinitesimal deformations, and from which most materials will deviate as deformations become finite. For example, Hencky elasticity reduces to linear elasticity for infinitesimal deformations; that is, equations (2.17) and (2.18) reduce to equations (2.20) and (2.21), respectively, for *u*_s_/*r*≪1 and ∂*u*_s_/∂*r*≪1. Alternatively, linear elasticity can instead be viewed as an exact constitutive law for an idealized material, for which it would be valid for arbitrarily large deformations.

Equation (2.22) can be combined with equations (2.7) and (2.12a) to provide a PDE for *u*_s_. In what follows, we use ‘Hencky elasticity’ to refer to equations (2.17)–(2.19) and ‘linear elasticity’ to refer to equations (2.20)–(2.22).

#### (iii) Linear poroelasticity

We now combine linearized kinematics (§2a(iv)) with linear elasticity (§2b(ii)). This then allows us to write equation (2.22) directly in terms of *ϕ*_f_ using equation (2.16),

2.23 $$\frac{\partial p}{\partial r}\approx M\frac{\partial }{\partial r}\left(\frac{\phi_{f}-\phi_{f,0}}{1-\phi_{f,0}}\right).$$

Equation (2.16) can then be rewritten as a linear second-order parabolic PDE for *ϕ*_f_.

### (c) Permeability laws

The solid skeleton deforms through rearrangement of the pore structure, leading to changes in the porosity. This is then likely to alter the permeability of the material. For infinitesimal deformations, this effect is second-order in the deformation, and is therefore typically neglected. We consider the impact of this simplification by comparing results for constant permeability with results for deformation-dependent permeability. As in MacMinn *et al.* [27], we adopt a normalized Kozeny–Carman formula,

2.24 $$k(\phi_{f})=k_{0}\frac{{\left(1-\phi_{f,0}\right)}^{2}}{\phi_{f,0}^{3}}\frac{\phi_{f}^{3}}{{\left(1-\phi_{f}\right)}^{2}},$$

where *k*_0_≡*k*(*ϕ*_f,0_) is the reference permeability. Although not quantitatively appropriate for all materials, this relation captures the important qualitative behaviour that *k*(*ϕ*_f_) vanishes as *ϕ*_f_ vanishes and *k*(*ϕ*_f_) diverges as *ϕ*_f_ tends to one.

Note that many materials have a naturally anisotropic permeability. In addition, anisotropic deformations may lead to the emergence of anisotropic permeability. For example, fluid flow through the walls of a porous cylinder leads to compression in the radial direction and stretching in the azimuthal direction, which might be expected to reduce the azimuthal permeability while enhancing the radial permeability. We neglect natural anisotropy here for simplicity, and induced anisotropy is irrelevant under the requirement of axisymmetry.

### (d) Initial state and boundary conditions

Before injection, the porosity is uniform, *ϕ*_f_(*r*,0)=*ϕ*_f,0_, the fluid and the solid are at rest, *v*_f_(*r*,0)=*v*_s_(*r*,0)=0, and the material is relaxed, $\sigma_{r}^{\prime }(r,0)=\sigma_{\theta }^{\prime }(r,0)=0$. We take this initial state to be the reference state, such that *u*_s_(*r*,0)=0.

#### (i) Injection

For *t*>0, we assume that fluid is injected from the origin either at an imposed constant volume flow rate per unit length $\hat{Q}$ or via an imposed constant pressure drop Δ*p*≡*p*(*a*,*t*)−*p*(*b*,*t*). It is straightforward to enforce the former condition since $\hat{Q}$ appears explicitly in the PDE. Enforcing the latter condition is less straightforward (electronic supplementary material, §B).

#### (ii) Inner boundary

The inner boundary is mechanically free, thus the normal effective stress must vanish. The inner boundary is also a material boundary. Hence, the appropriate mechanical and kinematic conditions are

2.25 $$\sigma_{r}^{\prime }(a,t)=0,\;u_{s}(a,t)=a(t)-a_{0}\;\mathrm{and}\;v_{s}(a,t)={\frac{\partial u_{s}}{\partial t}|}_{r=a}=\frac{da}{dt}.$$

#### (iii) Outer boundary

We consider two distinct sets of conditions at the outer boundary. In both cases, we assume without loss of generality that the fluid pressure vanishes at the outer boundary

2.26 p(b,t)=0.

If the outer boundary is subject to an applied effective stress, then this is a moving boundary. The appropriate mechanical and kinematic conditions are

2.27 $$\sigma_{r}^{\prime }(b,t)=\sigma_{r}^{⋆},\;u_{s}(b,t)=b(t)-b_{0}\;\mathrm{and}\;v_{s}(b,t)={\frac{\partial u_{s}}{\partial t}|}_{r=b}=\frac{db}{dt}.$$

Three conditions are required because the outer radius *b*(*t*) is unknown, and must be determined as part of the solution.

Alternatively, if the outer boundary is constrained such that its position is fixed, then the appropriate conditions are

2.28 $$u_{s}(b,t)=0\;\mathrm{and}\;v_{s}(b,t)={\frac{\partial u_{s}}{\partial t}|}_{r=b}=0.$$

This scenario requires only two conditions because the outer radius *b* is fixed and known. The normal component of the effective stress at the outer boundary $\sigma_{r}^{\prime }(b,t)$ is unknown, but does not need to be determined as part of the solution.

Conditions (2.28) are convenient for comparison with experiments and numerical simulations [29], and are relevant to industrial applications such as filtration. Conditions (2.27) are likely to be more relevant to biomedical and geotechnical applications.

#### (iv) Linearized boundary conditions

For the kinematically rigorous models, conditions at the inner and outer boundaries (equations (2.25)–(2.28)) are applied at *a*(*t*) and *b*(*t*), respectively. For the kinematically linearized models, these are instead applied at *a*_0_ and *b*_0_, respectively (e.g. $\sigma_{r}^{\prime }(a,t)=0\mapsto \sigma_{r}^{\prime }(a_{0},t)\approx 0$).

### (e) Non-dimensionalization and parameters

To proceed, we non-dimensionalize via the scaling

2.29 $$\tilde{r}=\frac{r}{b_{0}},\;{\tilde{u}}_{s}=\frac{u_{s}}{b_{0}},\;\tilde{a}=\frac{a}{b_{0}},\tilde{b}=\frac{b}{b_{0}},\;{\tilde{\sigma }}_{i}^{\prime }=\frac{{\sigma_{i}}^{\prime }}{M},\;\tilde{t}=\frac{t}{T_{\mathrm{pe}}}\;\mathrm{and}\;\tilde{p}=\frac{p}{M},$$

where $T_{\mathrm{pe}}\equiv b_{0}^{2}\mu /k_{0}M$ is the characteristic poroelastic timescale. We can then rewrite equation (2.12a) in dimensionless form,

2.30 $$\frac{\partial \phi_{f}}{\partial \tilde{t}}+\frac{1}{\tilde{r}}\frac{\partial }{\partial \tilde{r}}\left(\phi_{f}q(\tilde{t})-\tilde{r}(1-\phi_{f})\tilde{k}(\phi_{f})\frac{\partial \tilde{p}}{\partial \tilde{r}}\right)=0,$$

where $\tilde{k}(\phi_{f})=k(\phi_{f})/k_{0}$. Injection is characterized either by a fixed dimensionless flow rate *q* or by a fixed dimensionless pressure drop $\Delta \tilde{p}$,

2.31 $$q\equiv \frac{\mu \hat{Q}}{2\pi k_{0}M}\;\mathrm{or}\;\Delta \tilde{p}\equiv \frac{\Delta p}{M},$$

where, in the latter case, $q(\tilde{t})$ must be calculated from $\Delta \tilde{p}$ as part of the solution. Both of these quantities compare the characteristic pressure due to injection with the characteristic elastic stiffness of the material. The model is additionally characterized by the value of *ϕ*_f,0_ and three other dimensionless parameters:

2.32 $$\Gamma \equiv \frac{\Lambda }{M},\;{\tilde{a}}_{0}\equiv \frac{a_{0}}{b_{0}}\;\mathrm{and}\;{\tilde{\sigma }}_{r}^{⋆}\equiv \frac{\sigma_{r}^{⋆}}{M},$$

where *Γ* compares the bulk modulus to the shear modulus ($\Gamma \in [-\frac{1}{2},1]$, where *Γ*=1 corresponds to an incompressible material).

We work in dimensionless quantities from here onwards; hence, we drop the tildes for convenience.

### (f) Summary of models

Thus far, we have developed several different models for the response of a poroelastic cylinder to radially outward flow by considering two different representations of the kinematics (linearized and rigorous), two different elasticity laws (linear and Hencky) and two different permeability laws (constant and Kozeny–Carman). We categorize these models as linear ‘L’ (linearized kinematics with linear elasticity), quasi-linear ‘Q’ (rigorous kinematics with linear elasticity) and nonlinear ‘N’ (rigorous kinematics with Hencky elasticity). For each of these, we consider both constant ‘*k*_0_’ and Kozeny–Carman ‘*k*_KC_’ permeability. We then have six combinations: L-*k*_0_, L-*k*_KC_, Q-*k*_0_, Q-*k*_KC_, N-*k*_0_ and N-*k*_KC_. Note that L-*k*_0_ is classical linear poroelasticity and N-*k*_KC_ is fully nonlinear poroelasticity; the other four models are intermediate between these two extremes. Note also that we do not combine linearized kinematics with Hencky elasticity because this is asymptotically inconsistent; linearizing the kinematics requires that *u*_s_/*r*≪ and ∂*u*_s_/∂*r*≪1, under which assumptions Hencky elasticity reduces to linear elasticity.

## 3. Steady-state solutions

We now seek solutions to the above models at steady state, for which the fluid velocity is steady (∂*v*_f_/∂*t*=0) and the solid is stationary (*v*_s_=0). Combining equations (2.8), (2.10) and (2.15), we have

3.1 $$\frac{d\sigma_{r}^{\prime }}{dr}+\frac{\sigma_{r}^{\prime }-\sigma_{\theta }^{\prime }}{r}=\frac{dp}{dr}=-\frac{q}{rk(\phi_{f})},$$

where *ϕ*_f_=*ϕ*_f_[*u*_s_(*r*)]. Combining this with an elasticity law, a permeability law and a kinematic relationship between *u*_s_ and *ϕ*_f_ then leads to a second-order ODE in *u*_s_ for all models. For linear elasticity (L and Q models), we combine equation (3.1) with equation (2.21) to arrive at

3.2 $$\frac{d^{2}u_{s}}{dr^{2}}+\frac{1}{r}\frac{du_{s}}{dr}-\frac{u_{s}}{r^{2}}=-\frac{q}{rk[\phi_{f}(u_{s})]}.$$

For Hencky elasticity (N models), we combine equation (3.1) with equation (2.18) to arrive at

3.3 $$\frac{d^{2}u_{s}}{dr^{2}}=\frac{\left(1-\lambda_{\theta }/\lambda_{r})[\ln(\lambda_{r})+\Gamma \ln(\lambda_{\theta })-\Gamma ]+(1-\Gamma )\ln(\lambda_{\theta }/\lambda_{r})-q\lambda_{r}\lambda_{\theta }/k[\phi_{f}(u_{s})\right]}{\lambda_{r}r\{1-[\ln(\lambda_{r})+\Gamma \ln(\lambda_{\theta })]\}},$$

where the stretches are defined in equation (2.4). Note that equations (3.2) and (3.3) are valid for any permeability law, boundary conditions and treatment of kinematics.

Thus, we have a boundary-value problem (BVP) comprising a second-order ODE (equation (3.2) or equation (3.3)) with two constraints at the inner boundary (equations (2.25)) and either three or four constraints at the outer boundary, depending on whether the outer boundary is fixed (equations (2.26) and (2.28)) or not (equations (2.26) and (2.27)). For the L-*k*_0_ and Q-*k*_0_ models, the ODE (equation (3.2)) can be solved analytically (electronic supplementary material, §C). For the L-*k*_0_ model, this provides the full solution to the problem. For the Q-*k*_0_ model, it remains to solve an implicit algebraic system for *a* and, depending on the outer boundary condition, for *b*. This can be implemented with standard numerical root-finding techniques. For the other four models, the ODE cannot be solved analytically and we instead solve it numerically using a Chebyshev spectral collocation method, as described in §3b.

### (a) Injection

An imposed flow rate *q* will lead to a steady-state pressure drop Δ*p*. The latter is not needed as part of the solution, but can be calculated readily via the integration of equation (2.12b), giving

3.4 $$\Delta p=q\int_{a}^{b}\frac{1}{rk(\phi_{f})}dr.$$

By contrast, an imposed pressure drop Δ*p* will lead to a steady-state flow rate *q* that must be calculated as part of the solution by rearranging equation (3.4). For constant permeability, this relationship becomes

3.5 $$\Delta p=q\ln\left(\frac{b}{a}\right).$$

Everything else being fixed, the same steady state can therefore be achieved by imposing either *q* or Δ*p*. Clearly, the geometry and boundary conditions will have a strong impact on the relationship between *q* and Δ*p*. We explore this relationship in the next section.

### (b) Numerical solution via Chebyshev spectral collocation

When the ODE cannot be solved analytically, it must instead be integrated numerically as a BVP. Here, we use a direct method based on Chebyshev spectral collocation (i.e. a Chebyshev pseudospectral method) [34–36]. That is, we solve the BVP and all constraints simultaneously using a dense Chebyshev pseudospectral differentiation matrix and Newton iteration (electronic supplementary material, §D). This approach is robust and accurate, and also allows for the straightforward incorporation of additional unknowns and constraints, such as solving the problem for an imposed pressure drop Δ*p* rather than for an imposed flow rate *q*. We generate the differentiation matrices using the suite of Matlab functions provided in Weideman & Reddy [37].

## 4. Results

We have developed steady-state solutions for six different models, each for two distinct outer boundary conditions—a fixed outer boundary (‘constrained’) and an applied effective stress $\sigma_{r}^{⋆}$ at the outer boundary (see §2f). As described in §2e, these models are characterized by five dimensionless parameters: *Γ*, a ratio of elastic constants; *ϕ*_f,0_, the initial porosity; *a*_0_, the ratio of the initial inner radius to the initial outer radius; $\sigma_{r}^{⋆}$, the applied effective stress; and either *q*, the flow rate, or Δ*p*, the pressure drop. To focus on the impact of model choice, boundary conditions and geometry, we adopt fixed values of *Γ*=0.4 and *ϕ*_f,0_=0.5 throughout the rest of the paper. Varying these two parameters across a moderate range of typical values does not lead to dramatic qualitative differences in the resulting behaviour. Similarly, we fix $\sigma_{r}^{⋆}=0$ (‘unconstrained’) for simplicity.

### (a) Model comparison

**Figure 2.**
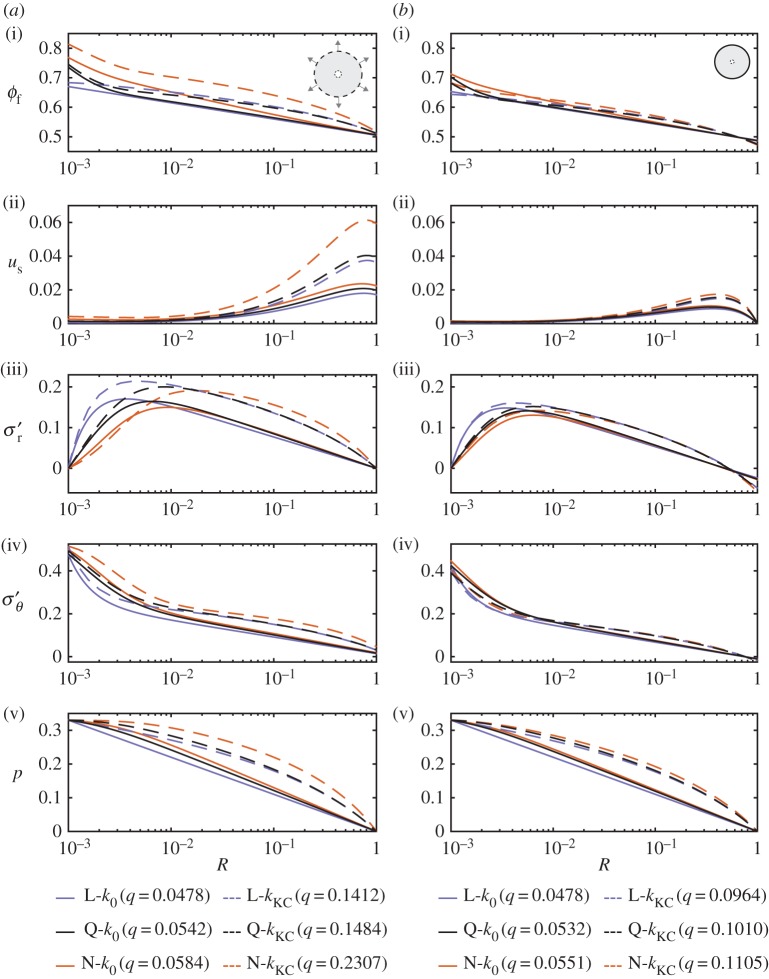
Six models at steady state for a thick-walled cylinder (*a*_0_=10^−3^). We consider (*a*) an unconstrained cylinder and (*b*) a constrained cylinder, both for flow driven by an imposed pressure drop Δ*p*=0.33. For clarity, we plot the results against the Lagrangian coordinate *R*(*r*,*t*)=*r*−*u*_s_ and on a logarithmic horizontal scale. The unconstrained and constrained cylinders exhibit very similar behaviour, implying that the distinction between these two outer boundary conditions is unimportant when the walls are very thick (i.e. for small *a*_0_). Additionally, note that in this case the permeability law has a stronger impact than the elasticity law or the treatment of the kinematics. (Online version in colour.)

**Figure 3.**
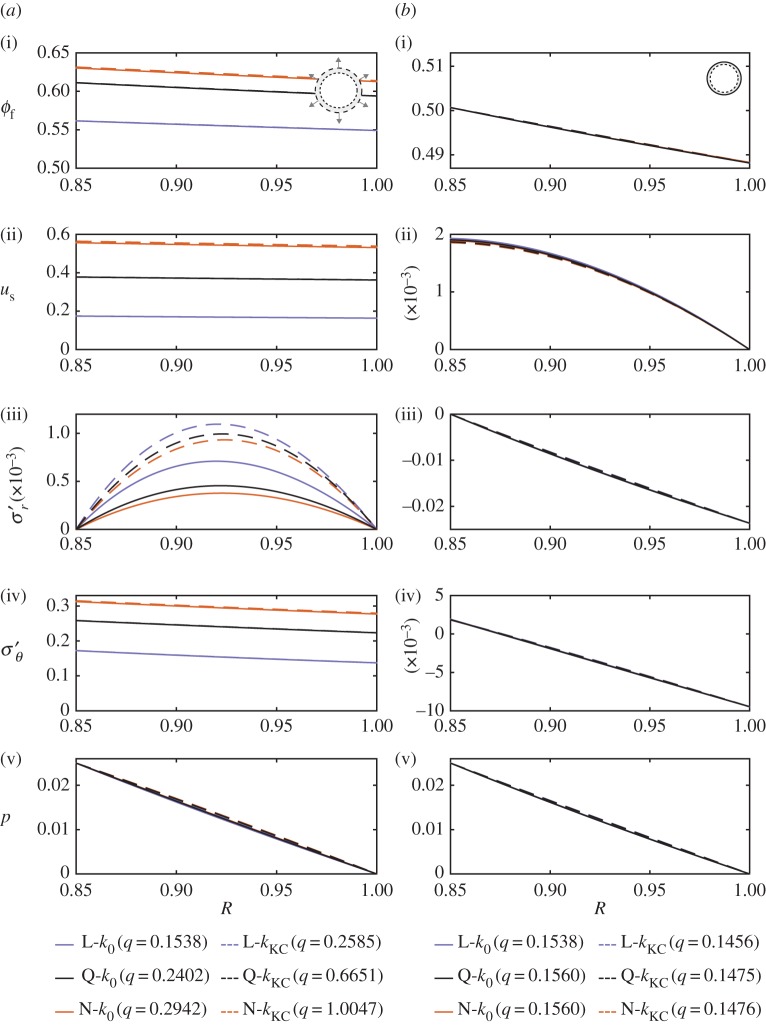
Six models at steady state for a thin-walled cylinder (*a*_0_=0.85). We again consider (*a*) an unconstrained cylinder and (*b*) a constrained cylinder, now for flow driven by an imposed pressure drop Δ*p*=0.025. For clarity, we plot the results against the Lagrangian coordinate *R*(*r*,*t*)=*r*−*u*_s_ on a linear horizontal scale. Unlike for the thick-walled cylinder (figure 2), the two different boundary conditions in this case result in strikingly different behaviour. For the unconstrained cylinder, the most important factors are the elasticity law and the treatment of the kinematics; the permeability law is relatively unimportant. For the constrained cylinder, all models exhibit nearly identical behaviour. (Online version in colour.)

In this section, we compare and contrast the six models for the two different boundary conditions (unconstrained and constrained) in the context of two end-member geometries: a thick-walled cylinder (figure 2) and a thin-walled cylinder (figure 3). This gives us a preliminary sense for how the geometry impacts the mechanics, which is in turn the focus of §4b.

#### (i) Unconstrained thick-walled cylinder

In figure 2, we consider a thick-walled cylinder for flow driven by an imposed pressure drop of Δ*p*=0.33. For an unconstrained thick-walled cylinder (figure 2*a*), the predictions of all models are qualitatively similar. The porosity *ϕ*_f_ (figure 2*a*(i)), azimuthal effective stress $\sigma_{\theta }^{\prime }$ (figure 2*a*(iv)) and pressure *p* (figure 2*a*(v)) all have maxima at the inner boundary and decrease monotonically from left to right. The porosity remains everywhere greater than *ϕ*_f,0_, the azimuthal effective stress is strictly tensile and the pressure drops from *p*(*a*,*t*)=Δ*p*=0.33 to *p*(*b*,*t*)=0 by construction. Additionally, the pressure profile is strongly nonlinear for the *k*_KC_ models, but closer to classical linear poroelasticity (L-*k*_0_) for the *k*_0_ models. In contrast to the behaviour of these quantities, the displacement *u*_s_ (figure 2*a*(ii)) and the radial effective stress $\sigma_{r}^{\prime }$ (figure 2*a*(iii)) are non-monotonic. The displacement has an interior maximum that is located in roughly the same place for all models. The radial effective stress vanishes at the inner and outer boundaries by construction. Between these limits, it is purely tensile with an interior maximum, with the location of this maximum depending strongly on model choice.

#### (ii) Constrained thick-walled cylinder

For the same pressure drop, a constrained thick-walled cylinder (figure 2*b*) exhibits a strikingly similar behaviour to that of the unconstrained cylinder. The maximum porosity at the inner boundary is lower than for the unconstrained cylinder, and the porosity now drops slightly below *ϕ*_f,0_ at the outer boundary where the material is slightly compressed. The displacement is qualitatively similar, but a factor of two to three smaller than in the unconstrained case. The radial and azimuthal effective stresses are now both slightly compressive at the outer boundary. This comparison between the unconstrained and constrained cylinders supports the intuition that the difference between these two cases becomes unimportant for thick walls (i.e. *a*_0_≪1).

In all of the cases shown in figure 2, the flow is driven by the same imposed pressure drop of Δ*p*=0.33. In addition to the above differences between the six models and the two boundary conditions, each of these 12 cases will result in a different flow rate^2^ *q* (see legend of figure 2). In all cases, *q* is lower for the constrained cylinder than for the unconstrained cylinder (again, except for the L-*k*_0_ model). This is because the inner radius of the constrained cylinder always expands less than that of the unconstrained cylinder, and *q* is very sensitive to the inner radius (equation (3.4)); the constrained cylinder is also slightly compressed against the outer boundary, which reduces its permeability in the *k*_KC_ models, amplifying the reduction in *q*.

All of the *k*_0_ models produce quantitatively similar values of *q*. For each, *q* differs by only a few percent between the two boundary conditions; between the *k*_0_ models for the same boundary condition, *q* differs by approximately 10–20%. By far the largest difference is between the corresponding *k*_0_ and *k*_KC_ models, where the *k*_KC_ model produces a value of *q* that is approximately two to four times larger than the corresponding *k*_0_ model. The permeability law makes a great difference since large deformations of a thick-walled cylinder lead to large and non-uniform changes in porosity. This substantial change in porosity leads to a substantial change in permeability for the *k*_KC_ models, but has no impact on the *k*_0_ models. This effect leads to higher values of *q* for the *k*_KC_ models because the average porosity is in all cases larger than *ϕ*_f,0_, so the permeability increases. Comparing the N models to the Q models, and the Q models to the L models, reveals that both rigorous kinematics and nonlinear elasticity also lead to higher values of *q* relative to their linearized counterparts. However, these effects are noticeably weaker than the impact of changing the permeability law. Given that the values of *q* vary so widely, it is surprising that the behaviour illustrated in figure 2 is otherwise so similar across the models and boundary conditions.

#### (iii) Unconstrained thin-walled cylinder

We now consider the other extreme geometry, a thin-walled cylinder, for a driving pressure drop of Δ*p*=0.025 (figure 3). Note that this value of Δ*p* is more than one order of magnitude less than the value used for the thick-walled cylinder (figure 2). Despite this much smaller value of Δ*p*, $\sigma_{\theta }^{\prime }$ here is comparable in magnitude to the thick-walled case while *u*_s_ is much larger. We discuss these points in more detail in §4b.

For the unconstrained thin-walled cylinder (figure 3*a*), *ϕ*_f_ (figure 3*a*(i)) is almost uniform across the domain, with a weak and roughly linear decrease from left to right. This behaviour is mirrored in *u*_s_ (figure 3*a*(ii)) and $\sigma_{\theta }^{\prime }$ (figure 3*a*(iv)). The pressure also decreases roughly linearly from left to right, from *p*(*a*,*t*)=Δ*p*=0.025 to *p*(*b*,*t*)=0, following classical linear poroelasticity for all models. Unlike for the thick-walled case, the permeability law is relatively unimportant for these quantities, whereas the kinematics and the elasticity law play much more prominent roles. Note that the kinematics consistently account for most of the difference between the L models and the N models (i.e. the Q models are closer to the N models than they are to the L models).

Unlike these other quantities, $\sigma_{r}^{\prime }$ does show a strong dependance on the permeability law. This suggests that the most direct impact of the permeability law is on $\sigma_{r}^{\prime }$, and this propagates to all other quantities when $\sigma_{r}^{\prime }$ is mechanically important (e.g. figure 2). For the unconstrained thin-walled cylinder, $\sigma_{r}^{\prime }$ vanishes at the boundaries and has an intermediate tensile maximum of order 10^−3^, whereas $\sigma_{\theta }^{\prime }$ is uniformly of order 10^−1^. As a result, the stark differences in $\sigma_{r}^{\prime }$ between the *k*_0_ and *k*_KC_ models are ultimately unimportant.

#### (iv) Constrained thin-walled cylinder

For the same pressure drop, the constrained thin-walled cylinder exhibits strikingly different behaviour to the unconstrained thin-walled cylinder. Whereas the unconstrained cylinder expands almost uniformly by 20–70%, the constrained cylinder is prevented from doing so. This results in much smaller displacements, with a maximum of order 10^−3^, making model choice essentially unimportant—all models approach their asymptotic limit of classical linear poroelasticity (L-*k*_0_). Note also that most of the material is in compression, with the porosity decreasing roughly linearly from a value just above *ϕ*_f,0_ at the inner boundary to a value noticeably below *ϕ*_f,0_ at the outer boundary. The displacement is weakly nonlinear, decreasing monotonically from left to right.

With regard to the flow rate *q*, we first note that the values of *q* in this case are substantially larger than the corresponding values for the thick-walled cylinder despite the fact that Δ*p* is much smaller. To rationalize this, note that the relationship between *q* and *a*_0_ for a given Δ*p* is strongly nonlinear even for a rigid cylinder (i.e. equation (3.5) with *a*=*a*_0_ and *b*=*b*_0_). The same is also true for classical linear poroelasticity, where the same expression also applies. In other words, this difference in *q* is due in large part to the fact that *a*_0_ is much larger.

For the constrained thin-walled cylinder, *q* is considerably smaller than for the unconstrained thin-walled cylinder (except for the L-*k*_0_ case, where *q* is independent of the boundary conditions). For the *k*_0_ cases, this is because the cylinder expands substantially and almost uniformly, which decreases the ratio of *b* to *a* and increases the flow rate (equation (3.5)). This is true to a much lesser extent for the constrained cylinder since the displacements are much smaller. For the *k*_KC_ models, this increase in *q* is substantially enhanced for the unconstrained cylinder by the noticeable increase in porosity and therefore permeability. The reverse occurs for the constrained cylinder, where the porosity decreases, leading a lower *q* for the *k*_KC_ models than for the *k*_0_ models. As for the thick-walled cylinder, both rigorous kinematics and nonlinear elasticity also lead to higher values of *q* relative to their linearized counterparts. For the unconstrained cylinder, these effects are substantial; for the constrained cylinder, these effects are noticeably weaker than the impact of the permeability law. There is relatively little difference in *q* across the six different models for the constrained cylinder, again because the displacements are necessarily small.

In this section, we have considered the implications of model choice in the context of two end-member geometries (thick- and thin-walled). We have shown that the error associated with linearization depends strongly on factors such as geometry and boundary conditions. In the next section, we study the mechanics of the problem over the full transition from *a*_0_≪1 to 1−*a*_0_≪1.

### (b) Impact of geometry

We now explore the parameter space more broadly, focusing on the importance of geometry (*a*_0_) and driving (*q* or Δ*p*) while again fixing *Γ*=0.4 and *ϕ*_f,0_=0.5. Although the N-*k*_KC_ model is arguably the most ‘correct’ of those considered above, it is much more computationally expensive than the other models. For simplicity, we restrict ourselves to the Q-*k*_KC_ model below. This model offers a good compromise between accuracy, robustness, and computational efficiency, demonstrating the same qualitative behaviour as the N-*k*_KC_ model for both end-member geometries and for both boundary conditions (electronic supplementary material, §F).

**Figure 4.**
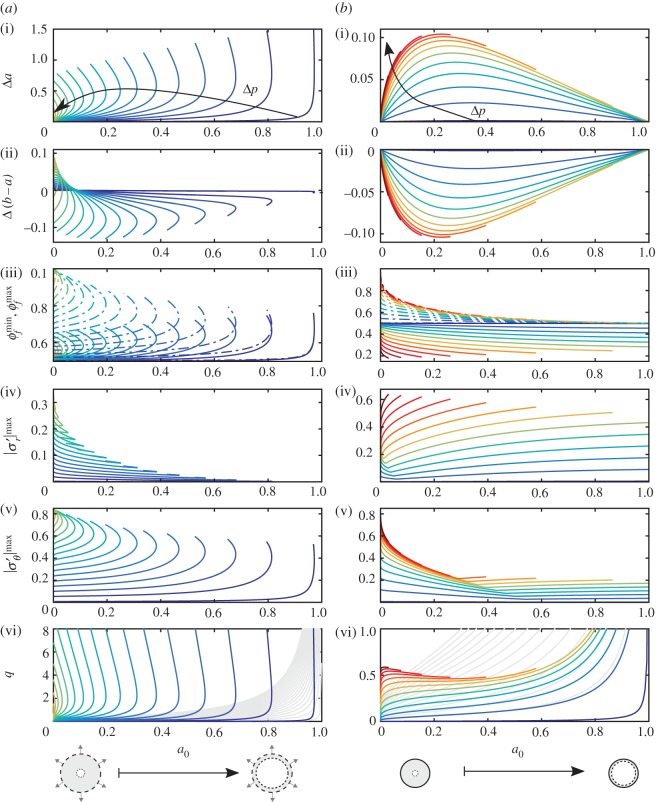
We explore the steady-state parameter space in more detail using the Q-*k*_KC_ model, plotting contours of fixed Δ*p* against *a*_0_ for several key quantities for (*a*) unconstrained cylinders (Δ*p*∈[0.005,0.5374], blue to yellow) and (*b*) constrained cylinders (Δ*p*∈[0.005,1.2], blue to red). We show (i) the change in inner radius Δ*a*; (ii) change in wall thickness Δ(*b*−*a*); (iii) minimum porosity $\phi_{f}^{\min}$ and maximum porosity $\phi_{f}^{\max}$ (solid and dot-dashed lines, respectively); (iv) maximum absolute radial effective stress $|\sigma_{r}^{\prime }{|}^{\max}$ and (v) maximum absolute azimuthal effective stress $|\sigma_{\theta }^{\prime }{|}^{\max}$; and (vi) flow rate *q*. We compare the latter with the reference flow rate *q*_0_ that would occur for a rigid cylinder with the same initial geometry, $q_{0}=\Delta p\ln{\left(b_{0}/a_{0}\right)}^{-1}$ (light grey lines). Note that the left and right columns use the same colour scale in Δ*p*. (Online version in colour.)

In figure 4, we consider the evolution of several key quantities as the inner radius *a*_0_ varies continuously from *a*_0_≪1 (thick walls) to 1−*a*_0_≪1 (thin walls). For a particular value of *a*_0_, the flow can be driven by imposing either a fixed pressure drop Δ*p* or a fixed flow rate *q*; the other quantity (*q* or Δ*p*, respectively) is then calculated as part of the solution.^3^ We drive the flow with a fixed pressure drop Δ*p* and plot the results for several values of Δ*p* for unconstrained cylinders (figure 4*a*) and constrained cylinders (figure 4*b*). The resulting flow rate *q* varies along these contours of fixed Δ*p* as shown in figure 4*a*(vi),*b*(vi).

Note that these same results can be presented in several different ways, which is useful for interpretation. Here, we show contours of fixed Δ*p* plotted against *a*_0_ (figure 4). In §E of the electronic supplementary material, we additionally show contours of fixed *q* against *a*_0_ (electronic supplementary material, figure E1), contours of fixed *a*_0_ against *q* (electronic supplementary material, figure E2) and contours of fixed *a*_0_ against Δ*p* (electronic supplementary material, figure E3).

#### (i) Unconstrained cylinders

For unconstrained cylinders (figure 4*a*), the most striking feature is the double-valued nature of all quantities for a certain range of *a*_0_. Specifically, our results suggest that there exists a Δ*p*-dependent maximum initial inner radius $a_{0}^{\max}(\Delta p)$, above which the problem appears to have no solution and below which the problem appears to have two distinct solutions for at least some range of *a*_0_. Although most of these contours terminate at some value of *a*_0_ beyond which our numerical scheme is no longer able to converge to a solution, the existence of complete branches for larger values of Δ*p* suggests that all contours would continue smoothly back to *a*_0_=0. For simplicity, we assume that this is indeed the case in the discussion below.

For $a_{0}>a_{0}^{\max}(\Delta p)$, no steady-state solution exists. This suggests that, for a given value of *a*_0_, there exists a maximum allowable driving pressure Δ*p*^max^(*a*_0_) that can be supported (electronic supplementary material, Fig. E3). This maximum is an inherent feature of poromechanical coupling in a radial geometry. In the absence of a change in constitutive behaviour, applying a pressure drop larger than Δ*p*^max^(*a*_0_) would lead to unbounded deformation and, ultimately, to material failure. The value of Δ*p*^max^ is finite and positive for 0<*a*_0_<1, diverging as *a*_0_ tends to zero and vanishing as *a*_0_ tends to one.^4^

For $a_{0}<a_{0}^{\max}(\Delta p)$, two distinct steady-state solutions exist for a given *a*_0_. These correspond to a less-deformed solution and a more-deformed solution, where the latter is characterized by more extreme values of all quantities except for $|\sigma_{r}^{\prime }{|}^{\max}$. This implies that a given Δ*p* can lead to one of two different flow rates for the same cylinder: a lower flow rate in the less-deformed state or a higher flow rate in the more-deformed state. The classical balloon-inflation problem in nonlinear elasticity famously also exhibits multiple solutions in certain regions of its parameter space; in that case, the effect is purely kinematic and nonlinear-elastic. Here, this effect results from the non-trivial coupling of kinematics and poromechanics, even for a linear elasticity law. In the remainder of this section, we focus on the characteristics of these two solutions.

Flow drives all parts of the material radially outward (*u*_*r*_>0 for all *r*), so that the inner and outer radii of the cylinder always increase, *a*>*a*_0_ and *b*>*b*_0_ (i.e. Δ*a*>0, figure 4*a*(i), Δ*b*>0, not shown). The wall thickness *b*−*a* may increase or decrease, depending on whether Δ*b* exceeds Δ*a* (Δ(*b*−*a*), figure 4*a*(ii)). For $a_{0}≳0.1$, both solutions are characterized by a decrease in wall thickness. For $a_{0}≲0.1$, the less-deformed solution instead corresponds to an increase in wall thickness. For $a_{0}≲0.01$, both solutions correspond to an increase in wall thickness.

For all values of *a*_0_ and Δ*p*, both the minimum porosity *ϕ*^min^_*f*_ and the maximum porosity *ϕ*^max^_*f*_ exceed *ϕ*_f,0_ (figure 4*a*(iii); solid and dot-dashed lines, respectively). This implies that the porosity increases throughout the material (*ϕ*_f_>*ϕ*_f,0_ for all *r*), which further implies that the total cross-sectional area always increases, regardless of whether the wall thickness increases or decreases. For sufficiently small Δ*p*, there exists a value of *a*_0_ at which *ϕ*^min^_*f*_ and *ϕ*^max^_*f*_ intersect, implying the existence of a family of solutions with uniform porosity. The difference between *ϕ*^min^_*f*_ and $\phi_{f}^{\max}$ increases monotonically with Δ*p* such that this intersection no longer exists at high Δ*p* (electronic supplementary material, Fig. E3).

The maximum absolute azimuthal effective stress $|\sigma_{\theta }^{\prime }{|}^{\max}$ (figure 4*a*(iv)) and the maximum absolute radial effective stress $|\sigma_{r}^{\prime }{|}^{\max}$ (figure 4*a*(v)) are relevant to material failure. The azimuthal component increases monotonically with Δ*p* along the less-deformed solution branch; the radial component exhibits a more complex behaviour, but $|\sigma_{r}^{\prime }{|}^{\max}<|\sigma_{\theta }^{\prime }{|}^{\max}$ for all *a*_0_ and Δ*p* (electronic supplementary material, Fig. E3).

The flow rate *q* exhibits the same striking feature as most other quantities—a region $a_{0}>a_{0}^{\max}(\Delta p)$ characterized by no solution, and a region $a_{0}<a_{0}^{\max}(\Delta p)$ characterized by two solutions (figure 4*a*(vi); coloured lines). We compare the actual flow rate *q* with the reference flow rate *q*_0_ that would occur for the same Δ*p* for a rigid cylinder with the same initial geometry, $q_{0}=\Delta p\ln{\left(b_{0}/a_{0}\right)}^{-1}$ (figure 4*a*(vi); grey lines). This reference flow rate is equivalent to the prediction of classical linear poroelasticity (L-*k*_0_), and it diverges for all Δ*p* as *a*_0_ tends to one. Note that *q*>*q*_0_ for all *a*_0_ and Δ*p*—that is, a deformable unconfined cylinder will always conduct a higher flow rate than a rigid cylinder of the same initial geometry, and this is a nonlinear effect.

#### (ii) Constrained cylinders

Constrained cylinders exhibit qualitatively different behaviour (figure 4*b*)—a single solution exists for all values of *a*_0_, and all quantities vary monotonically with Δ*p*. Note that we expect unconstrained and constrained cylinders to approach the same limiting behaviour for *a*_0_≪1, as noted above in the context of figure 2.

The change in inner radius Δ*a* is strictly positive, tending to zero for both small *a*_0_ and large *a*_0_. In the former limit, this is because Δ*a* decreases with *a*_0_ for fixed Δ*p*; in the latter limit, this is because *b* is fixed and the material has nowhere to go. The change in wall thickness is equal and opposite to the change in inner radius, Δ(*b*−*a*)=−Δ*a*, and is therefore strictly negative. That is, the walls always get thinner. As a result, the cross-sectional area always decreases and the average porosity (and thus *ϕ*^min^_*f*_) must always be less than *ϕ*_f,0_. However, $\phi_{f}^{\max}$ is still always greater than *ϕ*_f,0_. The difference between *ϕ*^max^_*f*_ and *ϕ*^min^_*f*_ increases with Δ*p* (electronic supplementary material, Fig. E3) and is roughly constant with *a*_0_. For a thin-walled cylinder, $\phi_{f}^{\max}$ is close to *ϕ*_f,0_ while *ϕ*^min^_*f*_ is substantially below *ϕ*_f,0_. For a thick-walled cylinder, *ϕ*^min^_*f*_ is close to *ϕ*_f,0_ while $\phi_{f}^{\max}$ is substantially above *ϕ*_f,0_. Note that the latter scenario respects the constraint on the average porosity by virtue of the fact that the large porosities are localized to a small region near the inner radius while the rest of the cylinder (the vast majority) is weakly compressed. The azimuthal stress $|\sigma_{\theta }^{\prime }{|}^{\max}$ decreases with *a*_0_ for small *a*_0_ and increases gently with *a*_0_ for large *a*_0_, tending to a finite, nonzero value as *a*_0_ tends to one. The radial stress $|\sigma_{r}^{\prime }{|}^{\max}$ exhibits a similar trend, with the transition from decreasing to increasing occurring at a much smaller value of *a*_0_. For both stress components, this transition occurs at a corner that corresponds to a transition in the maximum absolute value of the stress from tensile near/at the inner radius (radial/azimuthal) to compressive at the outer radius (both).

The flow rate *q* is weakly non-monotonic in *a*_0_ for small *a*_0_ and large Δ*p*, implying that two different values of *a*_0_ can lead to the same combination of Δ*p* and *q*. Comparing the actual flow rate *q* to the reference flow rate *q*_0_ (rigid cylinder or L-*k*_0_ model, grey lines), we find that a constrained deformable cylinder will conduct a larger flow rate than a rigid cylinder if the walls are thick, but a smaller flow rate than a rigid one if the walls are thin; this is in contrast to an unconstrained deformable cylinder, which always conducts a larger flow rate than a rigid one. This effect is amplified as Δ*p* increases, but its magnitude is relatively modest; *q* decreases from a few tens of percent above *q*_0_ to a few tens of per cent below *q*_0_ over the full range of *a*_0_. For an unconstrained cylinder, by contrast, deformation dominates the flow rate as *a*_0_ approaches $a_{0}^{\max}$.

### (c) Force balance

**Figure 5.**
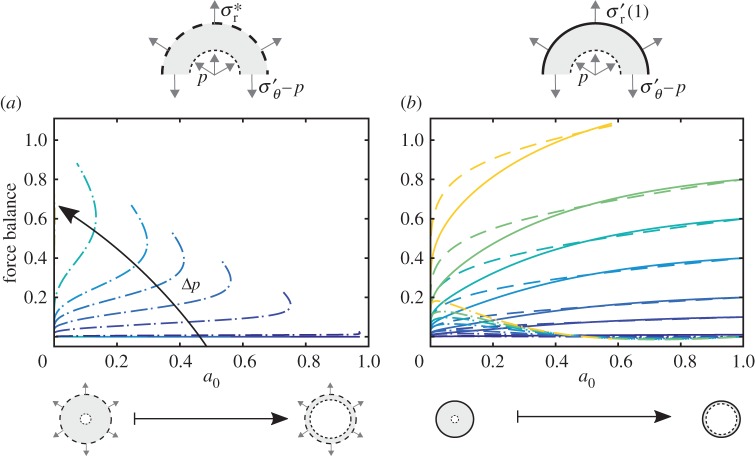
Flow leads to a net pressure force *F*_*p*_ (dashed lines) that must be supported by a combination of force due to internal azimuthal stress *F*_*θ*_ (dot-dashed lines) and force due to external radial traction *F*_*r*_ (solid lines). We plot these forces for (*a*) unconstrained cylinders (Δ*p*∈[0.005,0.5]) and (*b*) constrained cylinders (Δ*p*∈[0.005,0.6]). The colour scale for Δ*p* is the same as in figure 4. For unconstrained cylinders, note that *F*_*r*_≡0 and *F*_*θ*_≡*F*_*p*_. (Online version in colour.)

Flow always forces the material radially outward. This loading must be supported through a combination of internal azimuthal stress and external radial traction. To investigate these mechanics in more detail, we consider a macroscopic balance of the ‘vertical’ components of the forces acting on one-half of the annular cross-section of the cylinder (see diagrams, top of figure 5). The ‘vertical’ components of the forces due to fluid or pore-pressure loading *F*_*p*_, internal azimuthal stress *F*_*θ*_, and external radial traction *F*_*r*_ are given by

4.1 Fp=2aΔp+2∫abp dr,Fθ=2∫abσθ′ drandFr=−2bσr′(b)

and macroscopic force balance requires that *F*_*p*_=*F*_*θ*_+*F*_*r*_. We plot these quantities for unconstrained cylinders (figure 5*a*) and constrained cylinders (figure 5*b*). Note that, as with figure 4, these results can be presented in several different ways (electronic supplementary material, Fig. E4).

For unconstrained cylinders, *F*_*r*_≡0 and, therefore, *F*_*θ*_≡*F*_*p*_. These two non-trivial force components increase as Δ*p* increases along the less-deformed solution branch. These quantities ultimately mirror the behaviour shown in figure 4—two solutions exist for $a_{0}<a_{0}^{\max}(\Delta p)$, one corresponding to less deformation and smaller forces and the other corresponding to more deformation and larger forces.

For constrained cylinders, *F*_*r*_ will be determined implicitly to satisfy the condition that *u*_s_(1)=0. For fixed Δ*p*, both *F*_*p*_ and *F*_*r*_ increase monotonically with *a*_0_. For $a_{0}≲0.05$, *F*_*θ*_ is similar in magnitude to *F*_*r*_ and increases with *a*_0_; for $a_{0}≳0.05$, however, *F*_*θ*_ decreases rapidly with *a*_0_ and ultimately becomes weakly negative but negligible in the overall force balance. In other words, the outer boundary supports most of the fluid loading for a cylinder with moderate to thin walls. Note that *F*_*r*_<*F*_*p*_ for $a_{0}≲0.5$ since *F*_*θ*_>0, but *F*_*r*_>*F*_*p*_ for $a_{0}≳0.5$ since *F*_*θ*_<0.

## 5. Conclusion

Despite being a classical topic in geomechanics and in biophysics, radial poroelastic deformation has not previously been systematically explored, particularly in the context of large deformations. To assess the qualitative and quantitative impacts of large deformations, we considered six different models in the context of two end-member geometries (thick- and thin-walled) and two different outer boundary conditions (unconstrained and constrained). We showed that the impacts of nonlinear kinematics, nonlinear elasticity and deformation-dependent permeability depend strongly on geometry and boundary conditions, as does the relative importance of these facets of nonlinearity. For example, the mechanical response of an unconstrained thin-walled cylinder to an imposed pressure drop is dominated by kinematics and elasticity, although the permeability law exerts a strong control on the resulting flow rate through the material; for the same pressure drop, a constrained thin-walled cylinder is limited to much smaller deformations and exhibits what is essentially a linear-poroelastic response (figure 3). By contrast, the mechanical response of a thick-walled cylinder is much less sensitive to constraint, although the outer boundary condition has a strong impact on the flow rate when the permeability is deformation-dependent (figure 2).

To explore the importance of geometry and constraint in more detail, we then focused on a model that includes rigorous nonlinear kinematics and deformation-dependent permeability, but with the simplification of linear elasticity (Q-*k*_KC_). This model captures the qualitative and quantitative impacts of large deformations (electronic supplementary material, Fig. F1), but is more computationally convenient than a fully nonlinear model. We showed that, for an unconstrained cylinder, a given initial inner radius can conduct an arbitrarily large flow rate but can only support a finite maximum pressure drop, and this maximum allowable pressure drop increases with the thickness of the walls (figures 4 and 5; electronic supplementary material, figure E3). For a pressure drop less than this maximum, our results suggest that two valid solutions exist—a less-deformed state with a lower flow rate and a more-deformed state with a higher flow rate. A constrained cylinder, by contrast, can support an arbitrarily large pressure drop but can only conduct a finite maximum flow rate, and this maximum flow rate is non-monotonic in the wall thickness (electronic supplementary material, Figs E1–E3). These behaviours are mirrored in the corresponding force balances (figure 5; electronic supplementary material, figure E4).

We have assumed here that the constitutive response of the solid skeleton remains elastic for arbitrarily large deformations. This is relevant to biomedical applications such as fluid permeation through artery walls and to the design of radial filters. In geomechanical applications, however, large deformations are typically the result of material failure through plasticity or fracture, which will lead to a fundamentally different constitutive behaviour in the solid. Additionally, it may be relevant for many biomedical and geophysical applications to couple the poroelastic domain to different surface phenomena, such as free external flows. These behaviours may be the subject of future work.

We conclude by noting that, in addition to providing fundamental physical insight, our results and numerical codes (electronic supplementary material) could serve as a useful benchmark for general numerical-simulation tools (e.g. finite-element codes). Relatively few benchmarks are available in the context of large-deformation poroelasticity.

## Supplementary Material

Appendix

## Supplementary Material

MATLAB files

## Footnotes

1 In general, $\lambda_{i}^{2}$ are the eigenvalues of **F****F**^T^, where the superscript T denotes the transpose.

2 Except for the L-*k*_0_ model, for which *q* is independent of the boundary condition.

3 Note that one could instead impose both Δ*p* and *q* and calculate *a*_0_, which could be desirable in applications where *a*_0_ is a design parameter to be used for targeting a particular combination of Δ*p* and *q*. We do not consider this case here.

4 The limit *a*_0_→0 corresponds to a line source in an infinite domain, for which no steady state exists. The limit *a*_0_→1 corresponds to vanishingly thin walls, which can support no load.

## Data accessibility

The Matlab codes used to generate the results and figures are included in the electronic supplementary material.
