# Supplementary material for: From arteries to boreholes: steady-state response of a poroelastic cylinder to fluid injection
Source: Proc Math Phys Eng Sci. 2017 May 31;473(2201):20160753. doi: 10.1098/rspa.2016.0753 (PMC5454344; doi:10.1098/rspa.2016.0753)
Supplement: Appendix [file rspa20160753supp1.pdf]

Supplementary Appendix for

"From arteries to boreholes: Steady-state response of a poroelastic cylinder to fluid injection"

L. C. Auton and C. W. MacMinn

A. Hencky elasticity *vs.* linear elasticity for a uniaxial deformation

For a simple uniaxial deformation, Hencky elasticity reduces to

$$\frac{\sigma'}{\mathcal{M}} = \frac{\ln\lambda}{\lambda}, \tag{A1}$$

where σ' is the normal effective stress, $\mathcal{M}$ is the oedometric modulus, and $\lambda = 1 + \Delta L/L$ is the stretch, with ΔL the change in overall length and L the original length. Linear elasticity instead predicts

$$\frac{\sigma'}{\mathcal{M}} = \lambda - 1. \tag{A2}$$

We compare these behaviours in Figure A1.

B. Injection via fixed pressure drop

To enforce a constant pressure drop Δp, we must derive an associated expression for the evolving flow rate $q(t)$. To do so, we rearrange and integrate the expression for v_s from equation (2.12b) to

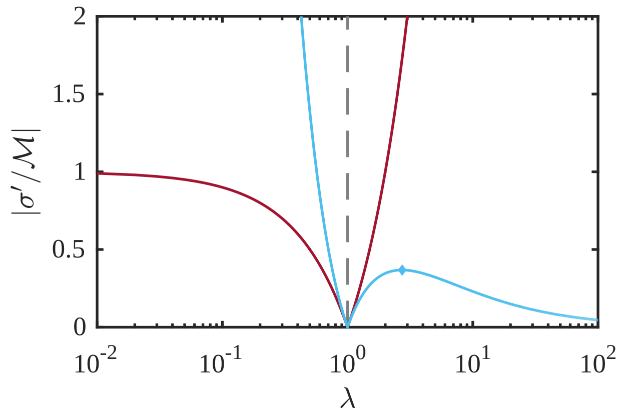

Figure A1. The absolute value of the dimensionless effective stress *vs.* the stretch for uniaxial deformation according to linear elasticity (red) and Hencky elasticity (blue). Hencky elasticity provides a stiffer response than linear elasticity in compression ($\lambda < 1$) and a softer response in tension ($\lambda > 1$). In tension, the stress predicted by Hencky elasticity reaches a maximum value of $\sigma'/\mathcal{M} = 1/e$ for a stretch of $\lambda = e$ (blue diamond) before decreasing asymptotically to zero. The two models agree in the limit of small strain, $|\lambda - 1| \ll 1$

obtain

$$q(t)=\frac{\Delta p+\displaystyle\int_a^b \frac{v_s}{k(\phi_f)}\,\mathrm{d}r}{\displaystyle\int_a^b \frac{1}{rk(\phi_f)}\,\mathrm{d}r} \tag{B1}$$

where

$$v_s=\frac{\mathrm{D}u_s}{\mathrm{D}t}=\lambda_r\frac{\partial u_s}{\partial t}. \tag{B2}$$

For linearised kinematics, we replace the latter expression with

$$v_s\approx\frac{\partial u_s}{\partial t}. \tag{B3}$$

The above expressions also apply at steady state, for which $v_s\equiv 0$.

C. Linear elasticity with constant permeability (L-k_0 and Q-k_0)

Assuming linear elasticity, we solve equation (3.2) for constant permeability ($k[\phi_f(u_s)]\equiv 1$) to arrive at a general expression for the displacement,

$$u_s=-\frac{qr\ln r}{2}+\frac{(2B_1+q)r}{2(1+\Gamma)}+\frac{B_2}{(1-\Gamma)r}, \tag{C1a}$$

where B_1 and B_2 are determined by the boundary conditions. This result is solely mechanical and constitutive, and is therefore valid for both the L-k_0 and Q-k_0 models. The general expressions for the effective stresses are then

$$\sigma_r'=-\frac{(1+\Gamma)}{2}q\ln r+B_1-\frac{B_2}{r^2}\quad\text{and}\quad\sigma_\theta'=-\frac{(1+\Gamma)}{2}q\ln r+B_1+\frac{B_2}{r^2}+\frac{q}{2}(1-\Gamma). \tag{C1b}$$

From these expressions, we arrive at four distinct solutions by combining the two different treatments of the kinematics (rigorous Q and linearised L) with the two different sets of outer boundary conditions (an applied stress at the outer boundary (equation 2.27) and a fixed outer boundary (equation 2.28)). The two L-k_0 solutions are classical solutions from linear poroelasticity [16]. An approximate version of the Q-k_0 solutions was derived by Barry and Aldis [10] and Barry and Mercer [11], who applied boundary conditions at the moving boundary but linearised the relationship between ϕ_f and u_s.

(a) Solution for L-k_0 with an applied effective stress at the outer boundary

For an applied effective stress at the outer boundary, we derive expressions for B_1 and B_2 by applying the appropriate inner and outer boundary conditions (equations (2.25) and (2.27), respectively). We linearise the kinematics by applying these at $r=a_0$ (rather than at a) and at $r=1$ (rather than at b), respectively. We obtain

$$B_1=\frac{2\sigma_r^\star-(1+\Gamma)q\ln(a_0)}{2(1-a_0^2)}+\frac{1+\Gamma}{2}q\ln(a_0)\quad\text{and}\quad B_2=\frac{a^2[2\sigma_r^\star-(1+\Gamma)q\ln(a_0)]}{2(1-a_0^2)}. \tag{C2}$$

(b) Solution for L-k_0 with a fixed outer boundary

Similarly, for a fixed outer boundary, we apply the appropriate inner and outer boundary conditions (equations (2.25) and (2.28), respectively) at $r=a_0$ and at $r=1$, respectively, to obtain

$$B_1=-(1-\Gamma)\left\{\frac{q[1+(1+\Gamma)\ln(a_0)]}{2[a_0^2(1+\Gamma)+(1-\Gamma)]}\right\}+\frac{(1+\Gamma)}{2}q\ln a_0 \tag{C3a}$$

and

$$B_2=-(1-\Gamma)\left\{\frac{qa_0^2[1+(1+\Gamma)\ln(a_0)]}{2[a_0^2(1+\Gamma)+(1-\Gamma)]}\right\}. \tag{C3b}$$

All other quantities can be derived from the expressions for u_s. Thus, we have complete explicit solutions following classical linear poroelasticity for the two different sets of outer boundary conditions. Note that, for linearised kinematics, ϕ_f should be calculated from u_s according to equation (2.16).

(c) Solution for Q-k_0 with an applied effective stress at the outer boundary

For an applied effective stress at the outer boundary, we now apply equations (2.25) and (2.28) at $r=a$ and $r=b$, respectively, to the general elastic solution (equation C1). This leads to

$$B_1 = \frac{b^2[2\sigma_r^\star + (1+\mathit{\Gamma})q\ln(b/a)]}{2(b^2-a^2)} + \frac{1+\mathit{\Gamma}}{2}q\ln(a), \quad B_2 = \frac{a^2b^2[2\sigma_r^\star + (1+\mathit{\Gamma})q\ln(b/a)]}{2(b^2-a^2)}. \tag{C4}$$

This solution is not explicit because the inner radius a and outer radius b are now determined by the two kinematic conditions (see equations (2.25) and (2.27)), leading to two coupled, implicit expressions for a and b. We solve these expressions numerically using a root-finding technique.

(d) Solution for Q-k_0 with a fixed outer boundary

For a fixed outer boundary, we now obtain

$$B_1 = -(1-\mathit{\Gamma})\left\{\frac{q[1+(1+\mathit{\Gamma})\ln(a)]}{2[a^2(1+\mathit{\Gamma})+(1-\mathit{\Gamma})]}\right\} + \frac{(1+\mathit{\Gamma})}{2}q\ln a \tag{C5a}$$

and

$$B_2 = -(1-\mathit{\Gamma})\left\{\frac{qa^2[1+(1+\mathit{\Gamma})\ln(a)]}{2[a^2(1+\mathit{\Gamma})+(1-\mathit{\Gamma})]}\right\}. \tag{C5b}$$

The problem is closed by applying the kinematic condition at the inner boundary (see equation (2.25)), leading to an implicit expression for a and the fixed outer boundary condition, equation (2.28). We again solve this numerically using a root-finding technique. As above, all other quantities can then be derived from the expressions for u_s. Note that, for rigorous kinematics, ϕ_f should be calculated from u_s according to equation (2.7).

D. Numerical solution via Chebyshev spectral collocation

When the ODE presented in §3 cannot be solved analytically, it must instead be integrated using standard numerical methods for BVPs, such as direct finite differences or a shooting method. For a shooting method, one must guess the locations of the free boundaries, solve the ODE as an initial value problem (IVP) subject to two of the constraints, and then iterate on the guesses until the remaining constraints are satisfied. For direct finite differences, two approaches are possible. One may follow the same approach as for a shooting method, but solving the BVP directly using finite differences and root finding (*e.g.*, Newton's method) rather than solving it as an IVP. Alternatively, one may solve the BVP and all constraints simultaneously using finite differences and root finding.

Although straightforward to implement, these approaches are unreliable in the present context because the iteration process can easily lead to a nonphysical state that prohibits further iteration. To mitigate these difficulties, we instead use a direct method based on Chebyshev spectral collocation (*i.e.*, a Chebyshev pseudospectral method) [*e.g.*, 35, 36]. That is, we solve the BVP and all constraints simultaneously as described above, but replacing the sparse finite-difference differentiation matrix with a dense Chebyshev-pseudospectral differentiation matrix. This approach still requires Newton iteration, but is more robust than finite differences because the density of the pseudospectral differentiation matrix directly couples the solution at each discrete point to the solution at every discrete point. This approach also allows for the straightforward incorporation of additional unknowns and constraints, such as solving the problem for an imposed pressure drop $\mathit{\Delta}p$ rather than for an imposed flow rate q. We illustrate

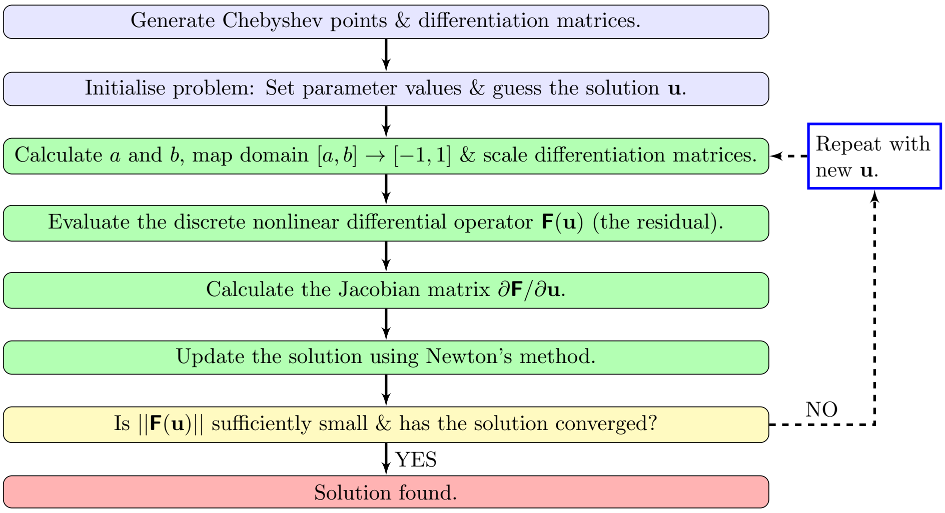

Figure D1. Procedure for direct solution via Chebyshev spectral collocation method.

the overall structure of the method in Figure D1. Note that, for purposes of Newton iteration, we calculate the Jacobian analytically for the L and Q models and numerically for the N models.

Spectral collocation methods involve discretising the solution domain into a set of N points (collocation points), defining a global function that interpolates the solution at these collocation points (the interpolant), and then approximating the derivatives of the solution as the derivatives of the interpolant. In a Chebyshev spectral collocation method, the collocation points are the N Chebyshev points $x_k \in [1, -1]$, which can be defined as [35]

$$x_k = \cos\left(\frac{(k-1)\pi}{N-1}\right), \quad k = 1, \ldots, N. \tag{D1}$$

The basis functions from which the interpolant is composed are then a set of N polynomials of degree $N-1$ satisfying the criterion that each is nonzero at exactly one distinct collocation point. Note that other definitions of the Chebyshev points are also commonly used [*e.g.*, 34]. For the definition given in equation (D1), Weideman and Reddy [37] provide a suite of `MATLAB` functions that generate the Chebyshev points and differentiation matrices, and that perform interpolation.

E. Impact of geometry

In Figures 4 and 5 of the main text, we plot the evolution of various key quantities as contours of fixed Δp against a_0. It is useful for interpretation to present the same results in several different ways. Here, we show the results of Figure 4 as contours of fixed q against a_0 (figure E1), contours of fixed a_0 against q (figure E2), and contours of fixed a_0 against Δp (figure E3). We also do the same for Figure 5 (figure E4).

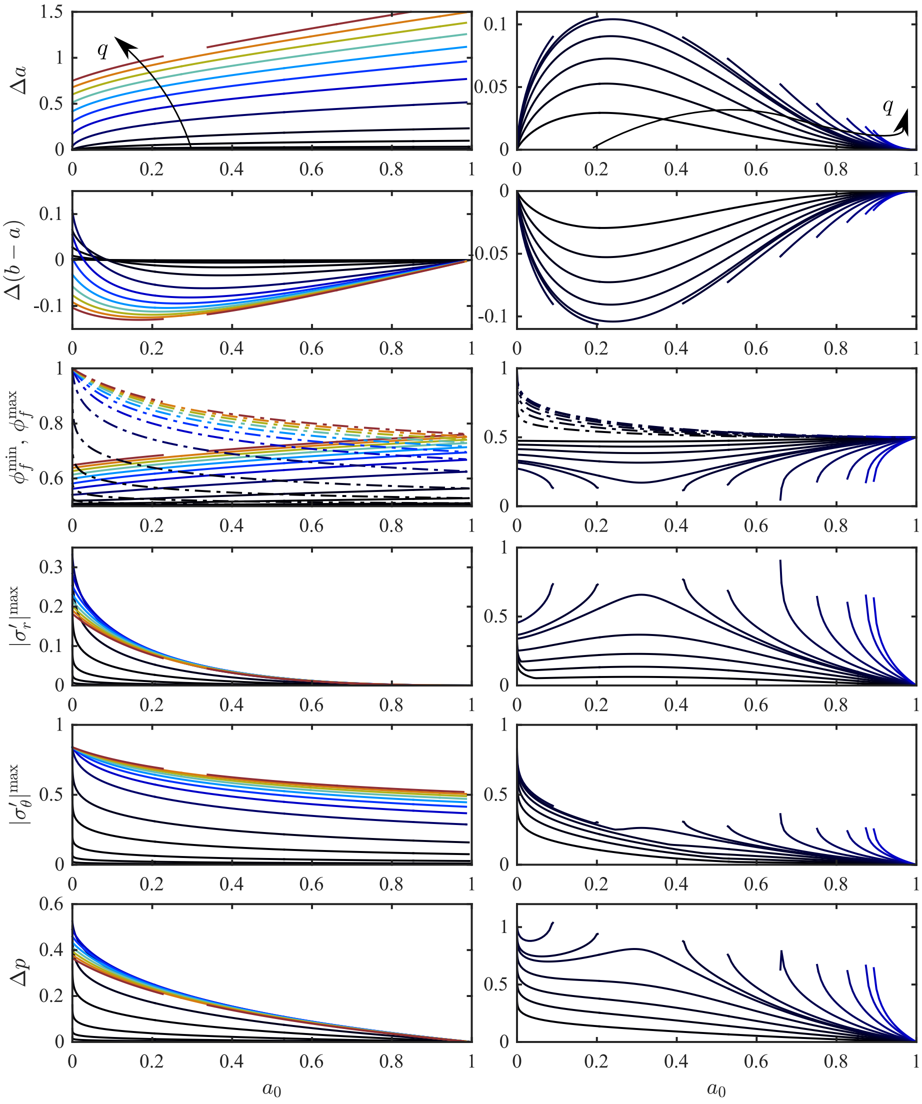

Figure E1. The results of Figure 4 plotted as contours of fixed q against a_0, with $q \in [0.001, 8]$ (left, black to red) and $q \in [0.001, 2]$ (right, black to blue).

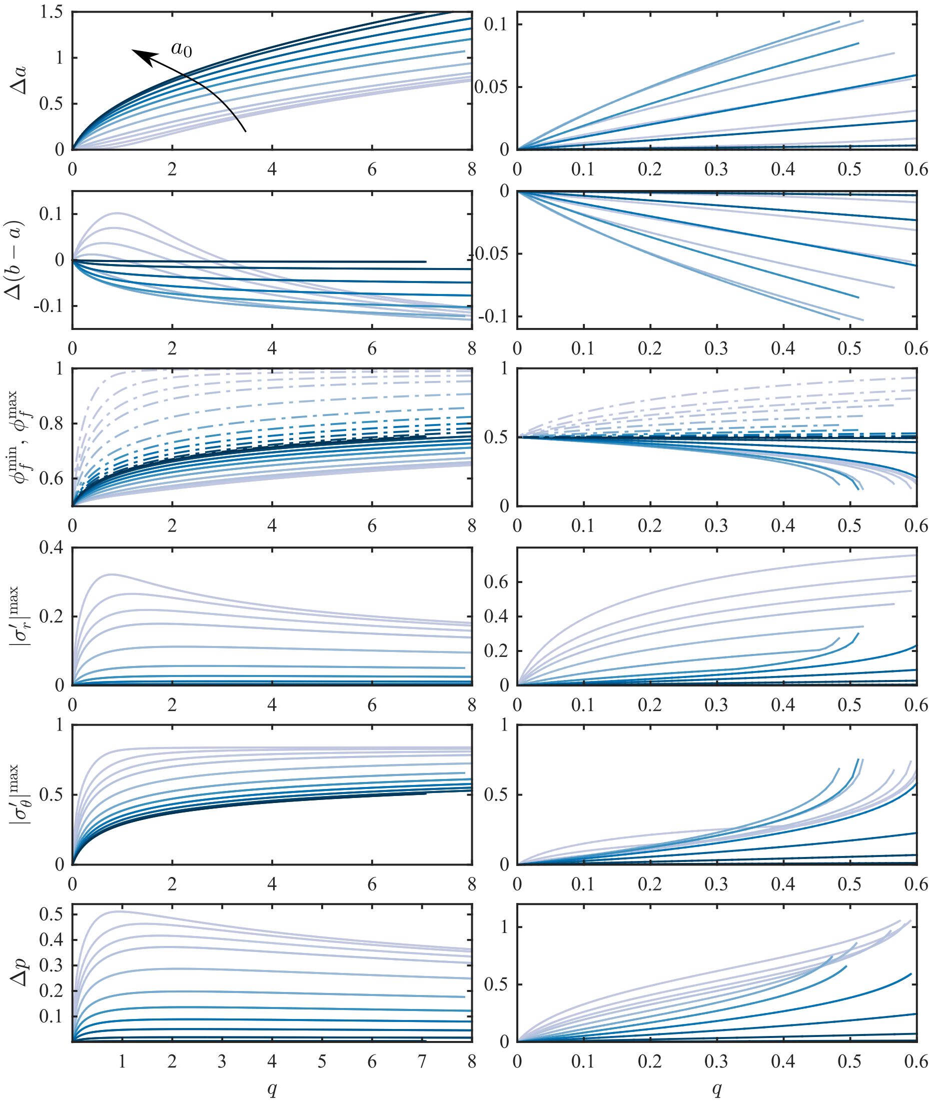

Figure E2. The results of Figure 4 plotted as contours of fixed a_0 against q, with $a_0 \in [0.001, 0.98]$ (light to dark).

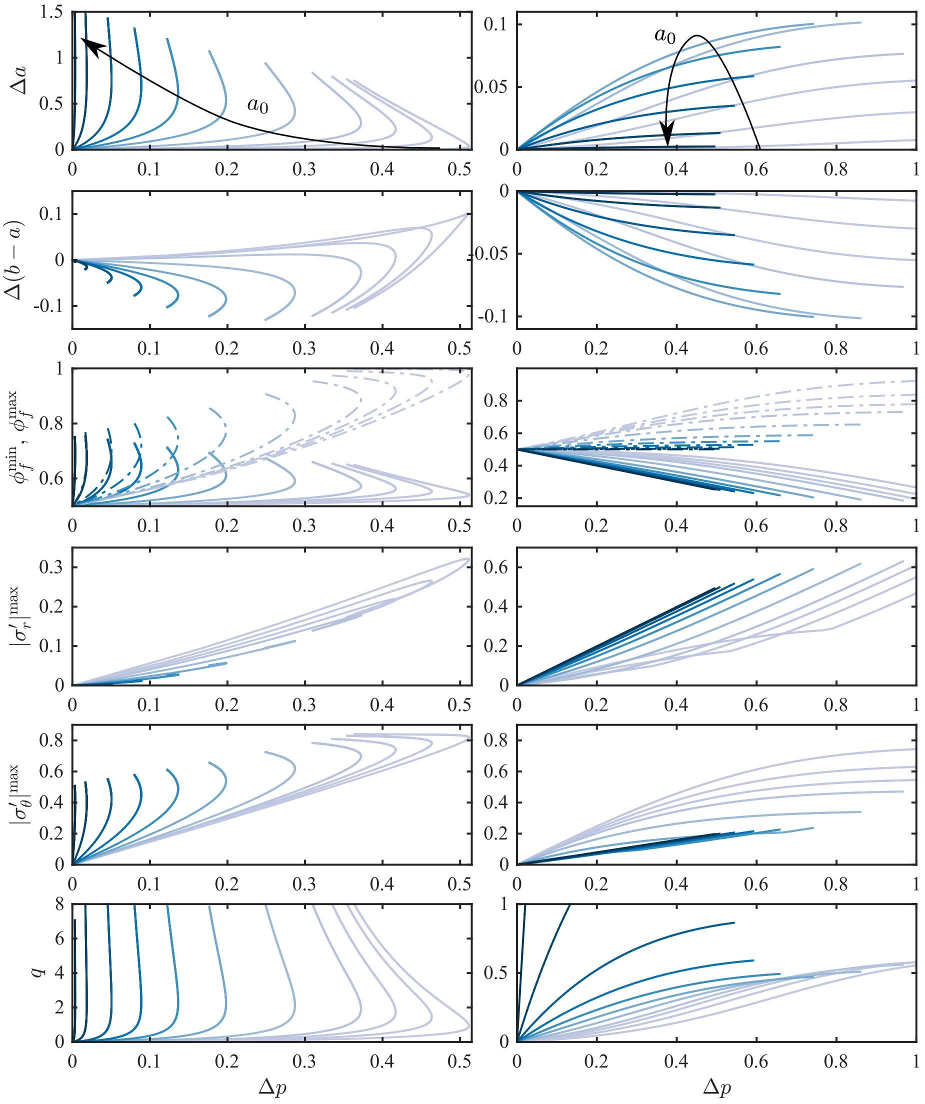

Figure E3. The results of Figure 4 plotted as contours of fixed a_0 against Δp, with $a_0 \in [0.001, 0.98]$ (light to dark).

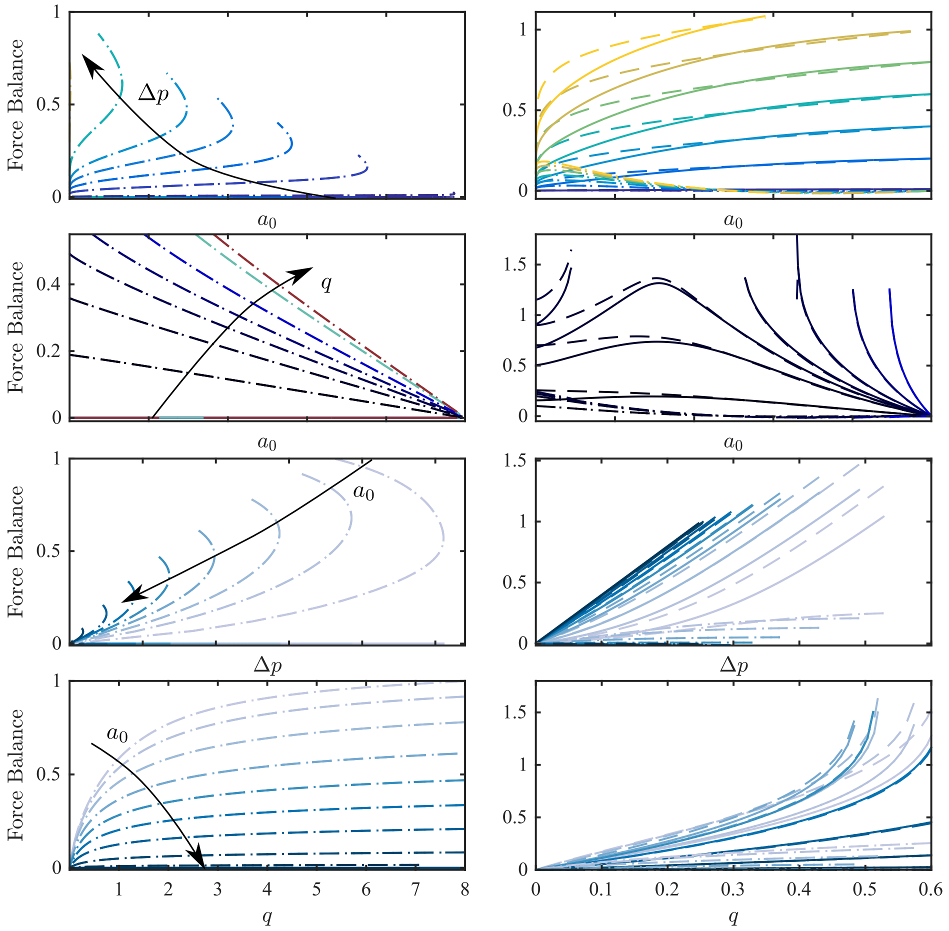

Figure E4. The results of Figure 5 plotted in various combinations. The colours in each row are the same as in the corresponding 'version' of figure 4.

F. Rheological effects

We focused in the main text on results from the Q-k_{KC} model because it provides a good compromise between rigour, robustness, and computational efficiency. We examine this choice in more detail in Figure F1 by plotting q against a_0 for contours of fixed Δp, as in the last row of Figure 4, for five different models: Q-k_{KC} (first row), L-k_{KC} (second row), Q-k_0 (third row), N-k_{KC} (last row), and L-k_0 (all rows, grey lines). Note that q appears to be much more sensitive to the permeability law than other aspects of the deformation (*cf.* Figures 2 and 3), making it a useful quantity for this comparison.

For unconstrained cylinders (left column), the Q-k_{KC} and N-k_{KC} models predict qualitatively similar behaviour, with the contours in the latter bending to the left somewhat more strongly. The latter model is also much more computationally expensive. The Q-k_0 model exhibits similar behaviour, but with much more extreme bending of the contours (note the different vertical scale). Our results for the L-k_{KC} model are inconclusive because this model is much less robust than either of the Q models; our method fails to find a solution for even moderate values of q. This is likely because the L-k_{KC} model is asymptotically inconsistent and does not correctly capture the kinematic relationship between porosity and displacement. The Q-k_{KC} is much more rigorous in these regards, and is only slightly more computationally expensive in our pseudospectral collocation framework.

For constrained cylinders (right column), all three of the k_{KC} models exhibit very similar behaviour despite the different elasticity laws (L and Q *vs.* N) and the different treatments of the kinematics (L *vs.* Q and N). This presentation does not constitutive a careful quantitative comparison, but it suggests that deformation-dependent permeability plays a key role in the mechanics of the problem, particularly for high pressures (right), whereas large-deformation kinematics are less important. This is somewhat unsurprising since constrained cylinders generally deform much less than unconstrained cylinders.

These results suggest that rigorous large-deformation kinematics (including the relationship between porosity and displacement) are important for model robustness and are central to the double-valued behaviour of unconstrained cylinders. Deformation-dependent permeability appears to moderate (but not eliminate) the double-valued behaviour of unconstrained cylinders, and to be central to the behaviour of constrained cylinders.

As noted in the main text, we have chosen Kozeny-Carman permeability and Hencky elasticity as relatively generic constitutive laws that capture the qualitatively important features of deformation-dependent permeability and large-deformation elasticity, respectively. Given the strong role of deformation-dependent permeability in our results, a comparison with results for other permeability laws would be an interesting topic for future work.

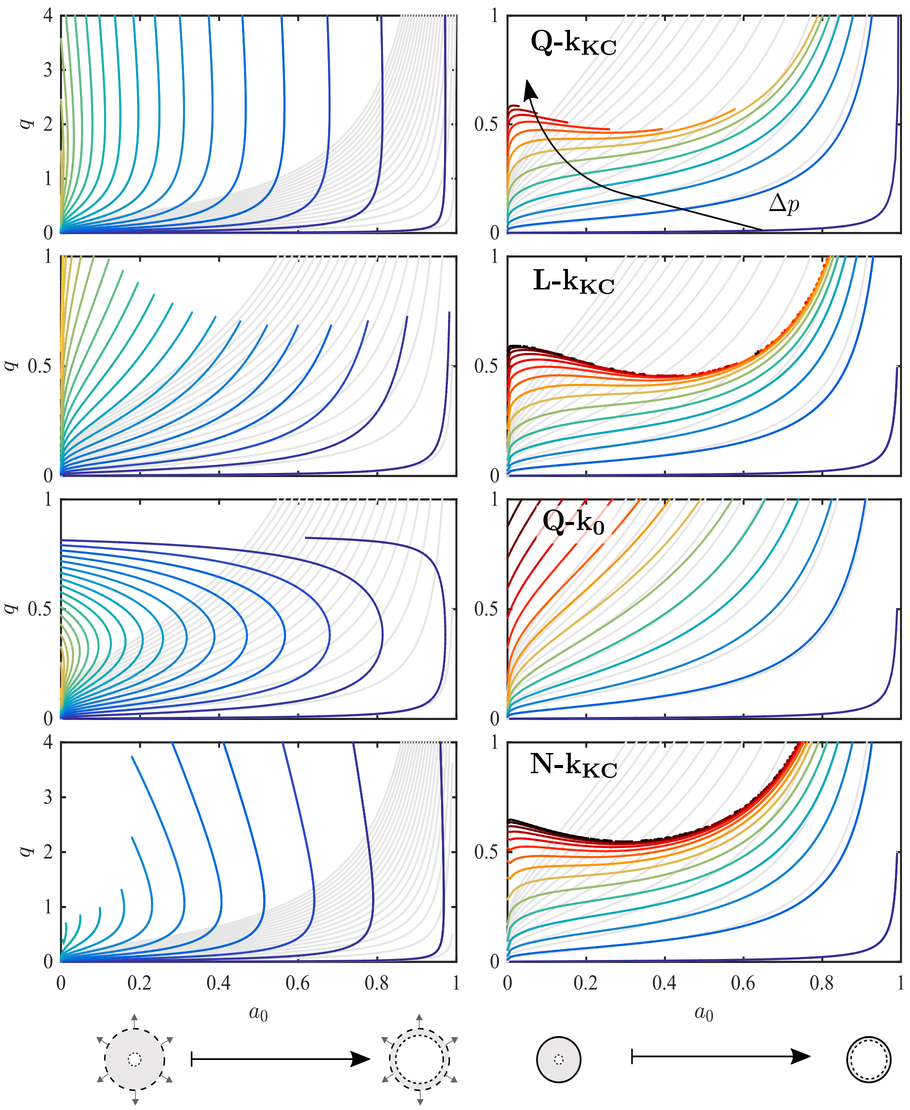

Figure F1. To illustrate the impact of rheology, we plot q against a_0 for contours of fixed Δp, as in the last row of Figure 4, for five different models: Q-k_{KC} (first row), L-k_{KC} (second row), Q-k_0 (third row), N-k_{KC} (last row), and L-k_0 (all rows, grey lines). The colour scale is the same as in Figure 4.

G. Solution for Q-k_0 in the thin-walled limit

We now derive an approximate solution to the Q-k_0 model in the limit of vanishing wall thickness, starting from equation (3.2) with $k[\phi_f(u_s)] \equiv 1$. We do this for the case of an applied effective stress at the outer boundary since the case of no displacement at the outer boundary is ultimately limited to small displacements and thus is well-captured by linear poroelasticity.

We begin by defining a new radial coordinate $\epsilon \equiv r - a$ such that $\epsilon \in [0, \delta]$ where $\delta \equiv b - a \ll 1$ is the wall thickness. We then rewrite equation (3.2) in terms of ϵ and seek a solution under the assumption that $\epsilon \ll 1$. From these assumptions, and writing $u_s(r) = U(\epsilon)$, we obtain at leading order

$$\frac{\mathrm{d}^2 U}{\mathrm{d}\epsilon^2} + \frac{1}{a}\frac{\mathrm{d}U}{\mathrm{d}\epsilon} - \frac{U}{a^2} = -\frac{q}{a}. \tag{G1}$$

Note that these assumptions require for asymptotic consistency that $q/a = O(1)$.

Equation (G1) is a linear, second-order ODE with solution

$$U = A_1 \exp\left[-\left(\frac{\sqrt{5}+1}{2a}\right)\epsilon\right] + A_2 \exp\left[\left(\frac{\sqrt{5}-1}{2a}\right)\epsilon\right] + aq. \tag{G2}$$

We now apply the relevant boundary conditions (equations (2.25) and (2.27) with $\sigma_r^\star \equiv 0$), which results in four equations for four unknowns: The two integration constant, A_1 and A_2, and the inner and outer radii, a and $a + \delta$, respectively. We use the two conditions at the inner boundary to derive expressions for A_1 and A_2 in terms of a,

$$A_1 = \frac{(a - a_0)(\Gamma - 1) + q}{\sqrt{5}} \quad \text{and} \quad A_2 = \frac{(\sqrt{5}+1)[a(1-q) - a_0] - \Gamma(a - a_0)}{\sqrt{5}}. \tag{G3}$$

The two conditions at the outer boundary then give

$$A_1 \exp\left[-\left(\frac{\sqrt{5}+1}{2a}\right)\delta\right] + A_2 \exp\left[\left(\frac{\sqrt{5}-1}{2a}\right)\delta\right] + aq = a + \delta - 1 \tag{G4a}$$

and

$$\left(\frac{\sqrt{5}-1}{2a}\right) A_2 \exp\left[\left(\frac{\sqrt{5}-1}{2a}\right)\delta\right] - \left(\frac{\sqrt{5}+1}{2a}\right) A_1 \exp\left[-\left(\frac{\sqrt{5}+1}{2a}\right)\delta\right] + \frac{\Gamma}{a}(a + \delta - 1) = \sigma_r^\star. \tag{G4b}$$

This is now a root-finding problem for the values of a and δ, which we solve numerically using the standard `MATLAB` function `fsolve`.

The pressure field is given by

$$\frac{\mathrm{d}p}{\mathrm{d}r} = -\frac{q}{r} \quad \mapsto \quad \frac{\mathrm{d}P}{\mathrm{d}\epsilon} = -\frac{q}{a} \quad \Longrightarrow \quad P(\epsilon) = \frac{q}{a}(\delta - \epsilon). \tag{G5}$$

This then leads to $\Delta p = (q/a)\delta$ and, since $q/a = O(1)$, we have that $\Delta p = O(\delta)$. This implies that a small pressure drop will drive a large flow rate when the walls are sufficiently thin.
